# A Web-Based Escape Room to Raise Awareness About Severe Mental Illness Among University Students: Randomized Controlled Trial

**DOI:** 10.2196/34222

**Published:** 2022-05-05

**Authors:** Jose M Rodriguez-Ferrer, Ana Manzano-León, Adolfo J Cangas, Jose M Aguilar-Parra

**Affiliations:** 1 Faculty of Psychology University of Almeria Spain

**Keywords:** escape room, severe mental disorder, higher education, nursing education, mental health, mental disorder, serious games

## Abstract

**Background:**

People with severe mental illness (SMI) face discriminatory situations because of prejudice toward them, even among health care personnel. Escape rooms can be a novel educational strategy for learning about and empathizing with SMI, thus reducing stigma among health care students.

**Objective:**

This study aimed to examine the effect of the Without Memories escape room on nursing students’ stigma against SMI.

**Methods:**

A pre- and postintervention study was conducted with a control group and an experimental group. A total of 306 students from 2 Andalusian universities participated in the study. Data were collected through a pre-post study questionnaire, consisting of an adapted version of the Attributional Style Questionnaire and a questionnaire on motivation for cooperative playful learning strategies. The control group carried out an escape room scenario without sensitizing content, whereas the experimental group carried out an escape room scenario on SMI, with both escape rooms being carried out in a 1-hour session of subjects related to mental health. To answer the research questions, a 2-way analysis of variance with repeated measures, a linear regression, and a 2-way analysis of variance were performed.

**Results:**

After the intervention, a significant reduction (*P*<.001) was observed in the experimental group in stigmatizing attitudes compared with the control group, in which no statistically significant changes (*P*>.05) were observed. In contrast, the linear regression (*t*_195_=−22.15; *P*<.001) showed that there was an inverse relationship between flow and the level of reduced stigma. When controlling for having or not having a close relative, the intervention was also shown to be effective (*P*<.001) in reducing the stigma displayed, both for people with affected and unaffected relatives.

**Conclusions:**

Our findings suggest that the Without Memories escape room can be used as an effective tool to educate and raise awareness about stigmatizing attitudes toward SMI in university students studying health care. Future testing of the effectiveness of educational escape rooms should be designed with new programs through playful strategies of longer duration to evaluate whether they can achieve a greater impact on motivation, acquisition of knowledge, and awareness. In addition, the feasibility of implementing the Without Memories escape room in other careers related to health and community should be investigated.

## Introduction

### Background

Severe mental illness (SMI) refers to different nosological entities with certain common severity criteria, lasting more than 2 years, and is associated with a loss of functionality [1]. Formally, it includes diagnoses such as schizophrenia, bipolar disorder, or severe personality disorders. The main difficulty faced by this group is stigma. People who are affected from stigma face discriminatory situations that prevent them from fully participating in society. Stigma is a multifactorial phenomenon that thrives on stereotypes and prejudices toward people with mental disorders. The most common stereotypes toward people with SMI are, for example, that they can be dangerous and commit more crimes than the general population [2]; they can be violent, unpredictable, and dependent; and they are responsible for their illness [2]. Stigma has severe consequences on the quality of life and self-esteem of people with mental disorders, hindering their social inclusion [3], which, in turn, has a negative effect on finding work, being economically independent, and having a solid circle of close friends and supporters [4].

To reduce stigma, different proposals for socioeducational programs have been made, including the dissemination of information, leisure activities such as sports or art, and contact with people with mental disorders. For example, in a meta-analysis study [5], through the analysis of 72 articles, it was observed that educational and social programs had positive effects in reducing stigma in adults and adolescents with mental illness.

With the arrival of serious games and educational video games, the possibilities of electronic resources in socioeducational intervention are also beginning to be studied. For example, in the video game Stigma-Stop, which aimed at reducing stigma toward people with mental disorders, its characters had various mental disorders, with whom participants could empathize. The results of 552 students aged between 14 and 18 years showed a statistically significant decrease in the levels of stigma toward people with schizophrenia [6].

Playful strategies, such as video games, have the potential to be widely motivating for students, making them pivotal to the acquisition of social skills among young people with mental disorders [7] and to the reduction of stigma [8].

In recent years, new forms of games have emerged and established themselves as a leisure alternative, including escape rooms [9]. Escape rooms consist of being locked in a room, solving a series of puzzles, unlocking locks, and finding hidden clues to escape from that room. Using a variety of settings and challenges, escape rooms create an experience that manages to be both motivating and educational for participants [10].

The results of recent studies [11] show that games and the use of escape rooms have been effective in engaging students in the learning process and helping them retain information. In higher education, specifically in the health care field, games have been used to evaluate the theoretical and practical knowledge of nursing students and study their motivation, where it can be observed that the students perceive that it helps them to learn, consider that these types of activities are fun, and believe that they should be included in their training curricula [12]. Owing to the situation caused by the COVID-19 pandemic, the daily face-to-face use of escape rooms has had to be transformed to apply it during web-based teaching. Previous studies [13-15] show that playful strategies have broad benefits in motivation and academic performance in massive web-based open courses.

### Objectives

University students in health care and social work studies are especially concerned that they are empathic with the population affected by mental disorders because they are one of the vulnerable groups in their professional future that they will have to attend to. The objective of this study was to evaluate the impact of a web-based escape room on raising awareness of SMI among university students in the health care branch during the 2020-2021 academic year in web-based mode. The specific objectives of this study were (1) to determine whether through a web-based escape room, it is possible to modify the stigmatizing attitudes of university students taking a nursing degree regarding SMI; (2) to study the influence of the immersion (flow) experienced during the escape room experience and on the awareness of the participants toward stigma; and (3) to determine whether the degree of stigmatizing attitudes is different between students with family members with severe mental disorders and students without family members with SMI and to analyze whether the program has the same effects on these people.

## Methods

### Participants

All the participants were first-semester nursing students. The participants were chosen based on the purpose of the research, consideration of the resources available when performing the intervention with web-based escape rooms, and the willingness of the university teachers to carry out the intervention in their class schedule.

**Figure 1:**
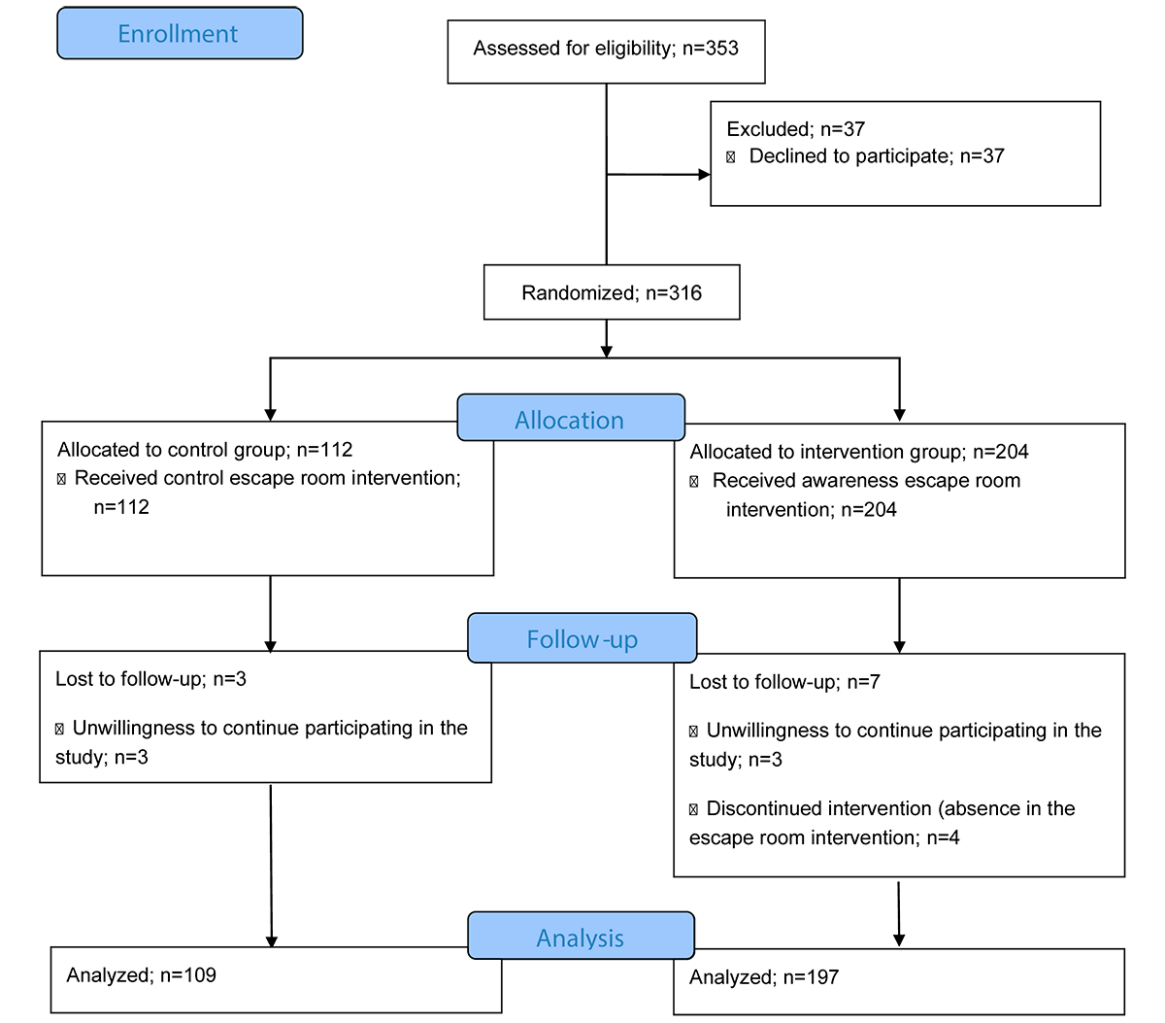
Flowchart followed for the selection of participants.

Both the escape room of the control group and the escape room of the experimental group were used during class time in subjects that had some content related to mental health. Students enrolled in the first semester of their nursing degree could participate in the research if they gave their written consent. The escape room did not score on the average of the course, and no financial reward or any other type of incentive was offered (Figure 1; Multimedia Appendix 1).

A total of 306 nursing students participated in the study, with a mean age of 23.34 (SD 8.37) years, comprising 57 (18.6%) men and 249 (81.4%) women. They were selected through cluster sampling, where a particular batch of students were selected as participants, dividing them by participating classrooms. They were randomly allocated between the control group and the experimental group. The experimental group consisted of 197 students with a mean age of 22.93 (SD 7.93) years, comprising 37 (18.8%) men and 160 (81.2%) women; the control group consisted of 109 students with a mean age of 24.1 (SD 9.10) years, comprising 20 (18.3%) men and 89 (81.7%) women.

To fulfill the third objective of this study—to determine whether the degree of stigmatizing attitudes is different between students with family members with severe mental disorders and students without family members with SMI and to analyze whether the program has the same effects on these people—demographic questions were asked of the students before beginning the intervention about whether they had relatives who had SMI. The data showed that 67 students had relatives with SMI, with a mean age of 22.78 (SD 4.27) years. Of these 67 students, 23 (34.3%) belonged to the control group, 19 (82.6%) women and 4 (17.4%) men, with a mean age of 23 (SD 4.62) years, and 44 (65.7%) belonged to the experimental group, 38 (86.4%) women and 6 (13.6%) men, with a mean age of 22.7 (SD 4.07) years.

### Ethics Approval

This study was conducted in accordance with the Declaration of Helsinki. All participants provided written informed consent. Ethics approval was obtained from the Research Ethics Committee of the University of Almería (Ref. UALBIO 2021/01).

### Instrument

#### Attributional Questionnaire

The 14-item abbreviated version of public stigma in mental health (Spanish) was used, adapted from the Attributional Questionnaire (AQ) 27 [16], with 14-point Likert-type questions from 1 to 9. It has a Cronbach α of .87. A higher score on this questionnaire indicates a greater number of stigmatizing attitudes toward people with SMI. It measures the following four factors:

1. Dangerousness—measures whether people with SMI pose a threat or create feelings of fear. An example question is, “Do you feel that people with SMI are dangerous?”
2. Solidarity—measures the willingness to help a person with SMI; for example, “If I owned an apartment, I would rent it to people with SMI.”
3. Coercion—people with SMI are required to participate in treatment. An example of an item for this factor is “I think it is in the best interest of the community where a person with SMI lives to be placed in a psychiatric facility.”
4. Avoidance—indicates the willingness to live or work near someone with SMI. For example, “I will share my car every day with a person with SMI.”

#### Motivation Questionnaire for Cooperative Playful Learning Strategies

The Motivation Questionnaire for Cooperative Playful Learning Strategies [17] was used to evaluate the learning process, degree of immersion, and motivation toward gamified activities. It is a Likert scale, ranging from 1 to 7, with 1 being totally disagree and 7 being totally agree. The flow factor has been used in this questionnaire, with a Cronbach α of .83. An example of the items contained in the factor is, “While playing, I was not aware of what was going on around me.”

For this study, an escape room has been designed based on SMI, namely, the *Without Memories* escape room. The narrative is that the main character must remember his or her identity and leave the apartment where he or she is to be on time for an appointment. In the escape room, the player wakes up with no memory of who or where he or she is and, through solving tests and exploration, discovers that he or she is a young man with a mental disorder. Through different elements, such as newspapers, mobile phones, social networks, and computers, a normalized life is gradually presented together with the different barriers that people with SMI face because of the stigma and discrimination they face; for example, difficulties in health services or difficulty finding work.

The escape room features linear mechanics: one test allows the next to be solved and so on until the final task, which requires the resolution of 2 tasks to obtain the code that ultimately allows the player to exit. The escape room is web-based and is designed within the *Genialy* digital platform, designed as a graphic adventure (Click and Point). The difficulty of the escape room is low because it is designed for a nonexpert audience in these activities, and its duration is approximately 1 hour.

### Procedure

To investigate whether web-based escape rooms could modify stigmatizing attitudes toward SMI, 2 web-based escape rooms were designed and evaluated according to the intervention group (Table 1).

**Figure 2:**
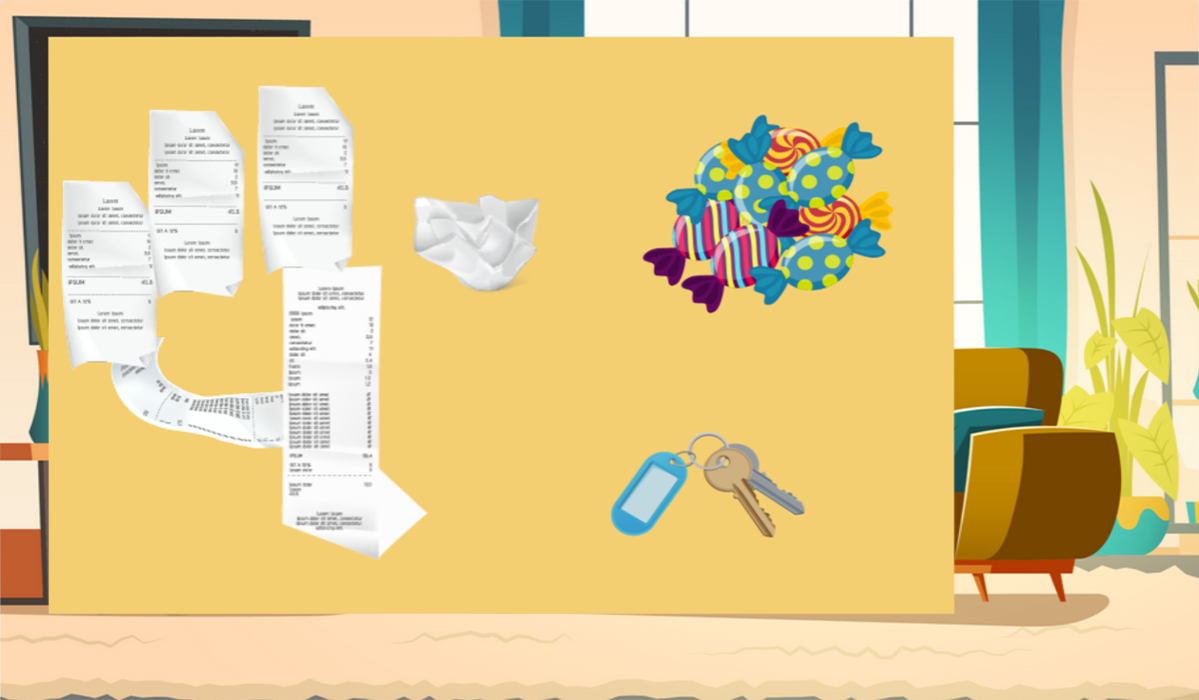
Example of elements where you must search for clues.

**Figure 3:**
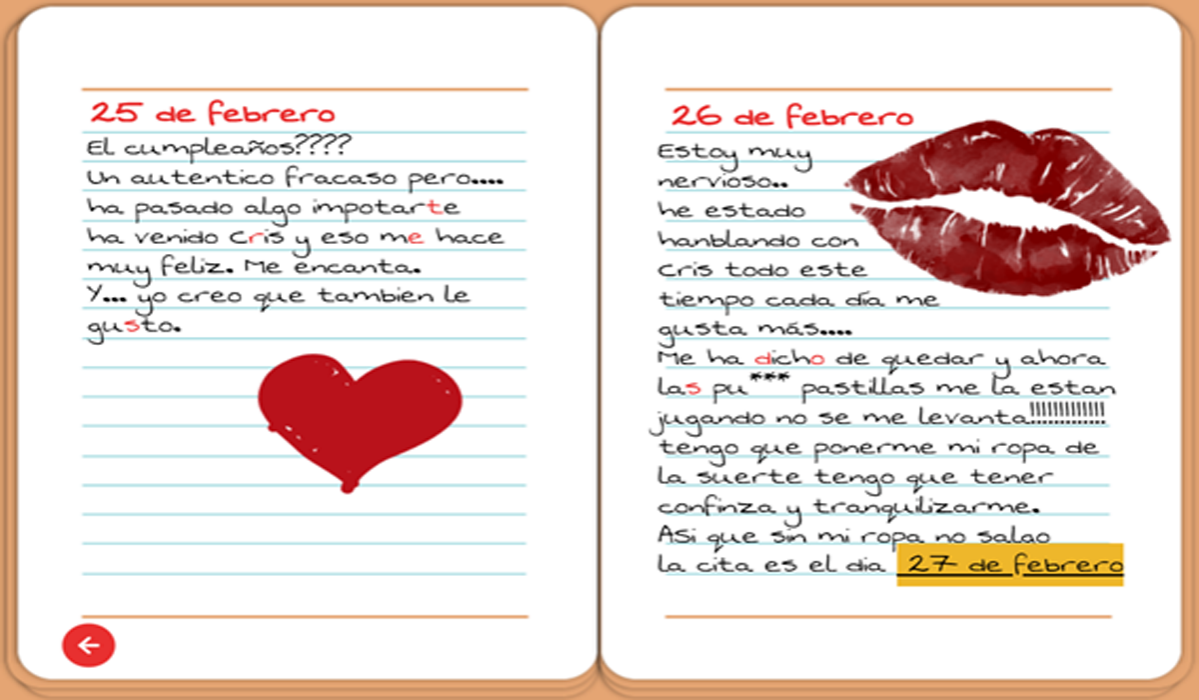
Example of awareness elements mixed with clues for other puzzles.

Owing to sanitary measures arising from the COVID-19 situation, both escape rooms were designed on the web. The escape rooms were prepared using the *Genially* digital platform, allowing the creation of *Click and Point* spaces, which is why it favors creating an interactive experience that can favor student immersion. To create the scenarios proposed in the escape room, images and illustrations from the FreePik resource bank were used, owing to a premium license for free use (Figures 2 and 3).

The main advantage of placing the escape room on the web is the ease of implementation in different universities through a link. The steps to carry out both escape rooms were as follows:

1. On the official platform of the university (Blackboard web-based learning platform), the class began, and the game master explained in a general way what an escape room was, how the Genially platform was used, and that this escape room was cooperative. Once any questions among the students had been resolved, the escape rooms were distributed to the students in teams of 4 people.
2. Each team was housed in a web-based workroom so that they could share ideas and opinions about solving the escape room. They had 2 options: either one of them shared a screen and they solved it together through that screen or each person solved the escape room simultaneously on their computer, maintaining the relationship between their partners in both options.
3. The game master was in the general room of the Blackboard platform during the entire escape room implementation time, in case a team contacted to request clues. There was no limit to the number of clues, as it was important that they felt they could receive feedback and were able to get out of the escape room even if they had never played before.
4. Once the team passed the last test and managed to leave the escape room, they completed the posttest questionnaire individually.

**Table 1:** Investigation procedure.

	Pretest	Intervention	Posttest
Control group	Attributional Questionnaire [14] and Motivation Questionnaire for Cooperative Playful Learning Strategies [16]	Escape room *Locked In*	Attributional Questionnaire [14] and Motivation Questionnaire for Cooperative Playful Learning Strategies [16]
Experimental group	Attributional Questionnaire [14] and Motivation Questionnaire for Cooperative Playful Learning Strategies [16]	Escape room *Without Memories*	Attributional Questionnaire [14] and Motivation Questionnaire for Cooperative Playful Learning Strategies [16]

To design the escape room *Without Memories*, interviews were conducted with people with SMI and 2 professionals from a local mental health association with the aim of understanding the difficulties people with SMI deal with daily and the reality they face as told from their own experiences. The game design for an escape room has a linear structure. Solving one clue will provide the object necessary to solve the next clue, and so on and so forth until the students escape. Tasks in a linear room must be solved in a particular order. The experiment was conducted on the web and synchronously. The average duration of the escape room was 60 minutes.

The escape room narrative involves waking up in a room without any memories of who or where you are. You must overcome different challenges until you know that your name is Enrique, you have an SMI, and you have a normal life.

The different tasks of the escape room are presented in the following formats inside the game:

- Computer: in this game, there are elements hidden around the room (click and point) that allow the student to know the password of a computer. On the computer, Enrique’s browsing history can then be accessed, where there are 2 videos of awareness campaigns on mental health and mental disorders, and Enrique’s social networks can be accessed, where 2 conversations with friends and a conversation with his family physician are observed, in which he mentions difficulties related to medication, such as the fear of not being able to have erections and to relate intimately with the woman he likes. Between these conversations, the key for the next test is observed.
- Diary and mobile phone: more conversations with Enrique’s family and friends are reflected on the mobile phone. These conversations reflect Enrique’s concerns and limited relationships. Some responses to his messages can be interpreted as condescension, childishness, and other behaviors that people with SMI reported in previous interviews. It is also narrated how these people may have a perception of the world that is different from the norm and may display different concerns and hobbies.
- Safe and door: the safe is opened by means of a phrase that is encoded in the computer. To achieve this, it is necessary to find the decoding key located in one of the rooms. This coded information allows the safe to be opened using information contained in the mobile phone and in the bedroom and allows you to remember that you are Enrique, you are an adult, you have an SMI, and you have met the girl you like for a coffee. The box finally gives you the key to open the door. When you click on the door again, there is a concluding message. This message provides statistical information about SMI and the possibility that the player may have an SMI or meet someone close with an SMI in the future.

For the control group, an escape room called *Locked In* was prepared. This escape room is a copy of the previous one in which all the elements that had been prepared to raise awareness, such as narrative texts and correspondence on social networks, were removed, replacing them with others without any burden of awareness, as they are logical tests, puzzles, and numerical locks. The narrative of this escape room assumes that you wake up in bed with no memories and must leave the apartment you are in. The steps for resolution are the same as those carried out by the experimental group; that is, they follow a linear logic in which one test leads to another until the final resolution is reached.

### Data Analysis

Initially, a sample size calculation was performed using the G*Power 3.1 program [18]. The parameters used for the calculation were α=.05 and statistical power (1−β)=0.80. For the effect size reference, the scientific literature was reviewed [6,14,19]. The effect size was moderate (*d*>0.20). Using these data, the minimum sample size needed for ANOVA testing was 277 students. Therefore, the sample size for this study was considered sufficient.

Before starting the analyses to answer the research questions, the direct scores of the administered tests were calculated using the corresponding descriptive statistics.

To answer the first research question, an ANOVA (2×2) with repeated measures was performed for each factor of the AQ14, as well as another test for the total result of the battery. The Bonferroni correction was used as the adjustment method for the comparison of post hoc tests.

To determine the relationship between flow and the change in stigmatizing attitudes, a linear regression was performed, in which the differential scores were calculated. In other words, pretest scores were subtracted from posttest measurements. With the differential scores and the score obtained in the flow, the linear regression was performed with the score of the flow as an independent variable.

To address the last research objective, a 3-way ANOVA was carried out for each of the factors and the total score of the AQ14.

The analyses were carried out using the R programing language with the R Studio development environment. The libraries used were ggplot2, tidyverse, emmeans, and rstatix.

## Results

The means and SDs are presented in Table 2. To better understand these results, it should be clarified that higher scores on the questionnaire indicate a greater stigma expressed by participants.

Regarding the first research question, ANOVAs (2×2) were performed with repeated measurements. The results of the tests between the groups are reported in Table 3, which shows the results of the ANOVAs along with the effect size using generalized eta squared. Post hoc tests are reported in this table, indicating the group in which there are statistically significant differences together with significance. This table shows that all statistical differences were found in favor of the experimental group in the posttest measurements. Table 4 indicates that the starting groups had equivalent scores, and statistically significant differences were found after applying the program. Regarding the effect sizes found in the variables, it can be indicated that in all the variables there is a large effect size (*η^2^_g_*>0.14), except in coercion, where the size is medium, and avoidance, where the size is small (*η^2^_g_*>0.01).

As for the second research question, as already mentioned, a linear regression was carried out between the flow variables (mean 18.4, SD 3.16), such as the independent variable, and the differential scores (mean −16.7, SD 18.4), such as the dependent variable. The results obtained in the analysis indicated that the predictor variables explained 84.5% of the total variance (*r^2^*=−0.845) and a moderate slope of the curve (*β*=−0.145; *t*_195_=−22.15; *P*<.001); that is, the higher the score obtained for flow, the more the stigma displayed was reduced.

**Table 2:** Mean and SD (pre- and posttreatment) of the control and experimental groups.

	Control, mean (SD)			Experimental, mean (SD)	
	Pretest	Posttest	Pretest		Posttest
Dangerousness	17.93 (6.78)	17.91 (6.76)	18.03 (7.57)		11.01 (6.17)
Solidarity	11.14 (5.5)	10.99 (5.39)	10.2 (5.48)		6.04 (3.58)
Coercion	8.63 (4.51)	8.87 (4.5)	8.18 (4.72)		5.65 (3.82)
Avoidance	11.86 (4.51)	11.78 (4.69)	11.16 (4.99)		8.13 (5.05)
Total	49.56 (16.03)	49.55 (16.02)	47.57 (16.7)		30.83 (14.79)

**Table 3:** ANOVA tests comparing between groups with the variables AQ14.

	Pretest				Posttest				Post hoc
	*F* test (*df*)	*P* value (adjusted)^a^	*η^2^_g_*	*F* test (*df*)		*P* value (adjusted)	*η^2^_g_*		
Dangerousness	0.01 (1,304)	.91	0	81.81 (1,304)		<.001	0.212	Exp^b^-Cont^c,d^	
Solidarity	2.04 (1,304)	.16	0.007	92.61 (1,304)		<.001	0.234	Exp-Cont^d^	
Coercion	0.67 (1,304)	.41	0.002	43.81 (1,304)		<.001	0.126	Exp-Cont^d^	
Avoidance	1.52 (1,304)	.22	0	38.60 (1,304)		<.001	0.011	Exp-Cont^d^	
Total battery	1.03 (1,304)	.62	0.003	106.16 (1,304)		<.001	0.258	Exp-Cont^d^	

^a^Bonferroni adjusted.

^b^Exp: experimental.

^c^Cont: control.

^d^*P*<.001.

**Table 4:** ANOVA tests comparing within groups with the variables AQ14.

	Control				Experimental				Post hoc
	*F* test (*df*)	*P* value (adjusted)^a^	*η^2^_g_*	*F* test (*df*)		*P* value (adjusted)	*η^2^_g_*		
Dangerousness	0.01 (1,108)	.90	0	196.13 (1,196)		<.001	0.206	Pre-post^b^	
Solidarity	2.12 (1,108)	.15	0	104.81 (1,196)		<.001	0.169	Pre-post^b^	
Coercion	3.68 (1,108)	.58	0	55.42 (1,196)		<.001	0.08	Pre-post^b^	
Avoidance	0.47 (1,108)	.49	0	64.61 (1,196)		<.001	0.084	Pre-post^b^	
Total battery	0.01 (1,108)	.98	0	163.19 (1,196)		<.001	0.221	Pre-post^b^	

^a^Bonferroni adjusted.

^b^*P*<.001.

The means and SDs before and after the intervention are displayed in Table 5, differentiating those who reported having a close relative and those who did not, both for the control group and the experimental group.

The results of the ANOVA indicated that having a close relative influences the level of stigma of the participants in all study variables (dangerousness: *F*_1.604_=6.33, *P*=.01, *η^2^_g_*=0.01; solidarity: *F*_1.604_=30.8, *P*<.001, *η^2^_g_*=0.04; coercion: *F*_1.604_=25, *P*<.001, *η^2^_g_*=0.04; avoidance: *F*_1.604_=49.5, *P*<.001, *η^2^_g_*=0.08; and total battery: *F*_1.604_=42.2, *P*<.001, *η^2^_g_*=0.06), with statistically significant differences in favor of people who have a close relative. In other words, people with a close relative with SMI display a lower level of stigma than those without a close relative with SMI.

With the intention of knowing how the program affects the fact of having or not having family members with SMI, the 3-way ANOVA was continued, and the results of this are reported for the posttest measures. The ANOVA results are presented in Table 6. In addition, the post hoc tests are reported, indicating only the statistically significant results.

As can be seen, statistically significant differences were found between the control and experimental groups, especially in the case of not having a family member affected by SMI. In this case, statistically significant differences were observed in all study variables. However, when someone had a family member affected by SMI, no statistically significant changes were found for the variables solidarity, coercion, and avoidance, whereas for the variables dangerousness and total battery, there were statistically significant changes between the people in the control and experimental groups who had family members affected by SMI.

**Table 5:** Means and SDs of participants with and without relatives with severe mental illness in the control and experimental group.

	Control-pre, mean (SD)		Experimental-pre, mean (SD)		Control-post, mean (SD)		Experimental-post, mean (SD)	
	Family	No family	Family	No family	Family	No family	Family	No family
Dangerousness	15.43 (5.68)	18.59 (6.92)	16.48 (7.59)	18.46 (7.53)	15.95 (6.03)	18.43 (9.92)	11.48 (5.95)	10.87 (6.24)
Solidarity	7.91 (4.36)	12 (5.47)	7.48 (4.52)	10.96 (5.49)	7.82 (4.03)	11.83 (5.40)	5.58 (3.43)	6.16 (3.62)
Coercion	6.39 (4.08)	9.23 (4.45)	5.81 (3.58)	8.83 (4.79)	6.69 (3.94)	9.45 (4.48)	4.60 (2.87)	5.94 (3.99)
Avoidance	8.30 (4.34)	12.81 (4.08)	7.6 (4.65)	12.14 (4.62)	8.34 (4.93)	12.69 (4.19)	6.90 (4.85)	8.46 (5.06)
Total	38.04 (15.23)	52.63 (14.86)	37.39 (16.70)	50.40 (15.61)	38.82 (15.90)	52.41 (14.86)	28.58 (12.56)	31.45 (15.33)

**Table 6:** Three-way ANOVA with the interaction between the control and experimental group and whether they have declared a close relative or not in posttest measures.

	Family			No family				Post hoc
	*F* test (*df*)	*P* value (adjusted)^a^	*η^2^_g_*	*F* test (*df*)	*P* value (adjusted)	*η^2^_g_*		
Dangerousness	6.41 (1,65)	<.001	0.011	67.512 (1,238)	<.001	0.101	Exp^b^-cont^c^ (no family)^d^/exp-cont (family)^e^	
Solidarity	3.317 (1,65)	.280	0.005	77.812 (1,238)	<.001	0.114	Exp-cont (no family)^d^	
Coercion	3.61 (1,65)	.232	0.006	37.40 (1,238)	<.001	0.058	Exp-cont (no family)^e^	
Avoidance	1.45 (1,65)	.916	0	46.028 (1,238)	<.001	0.071	Exp-cont (no family)^d^	
Total battery	6.79 (1,65)	.036	0.011	105.88 (1,238)	<.001	0.148	Exp-cont (no family)^d^/exp-cont (family)^f^	

^a^Bonferroni adjusted.

^b^Exp: experimental.

^c^Cont: control.

^d^*P*<.001.

^e^Persons without family members affected by severe mental illness.

^f^Persons with family members affected by severe mental illness.

## Discussion

### Principal Findings

This study sought to provide new methodologies to raise awareness among the young adult population, especially university students who in their professional future could work with people with severe mental disorders. There are very few studies that have used a web-based methodology [20], and at present, we do not know about any other study that has been based on an escape room, despite being a methodology that is being used more and more in the educational field. A recent systematic review on this topic did not identify any studies that applied this tool [21]. Applying them in a web-based format is also novel and, of course, was brought on by the effects of the pandemic, which resulted in a rapid adaptation to these methods [22].

However, as stated earlier, escape rooms are an increasingly used tool in the educational field [23,24] because of their playful contribution and proximity to the current language of young people.

In this study, we investigated the effectiveness of an escape room as an awareness tool based on empathizing or putting oneself in the role of a leading character with an SMI. The intervention significantly reduced the stigmatizing attitudes of university students in the experimental group compared with the control group. It is worth highlighting the size of the large effect observed in the factors of danger, perceived fear, and in solidarity, variables in which the greatest reduction was obtained.

Dangerousness is the most common stigma dimension in the general population [25], and it scored the highest in the pretreatment evaluation in this study. In contrast, because of the characteristics of the sample, the intervention also had a great effect on solidarity. More specifically, providing information about mental health problems in a participatory way, such as in the escape room, favored the strengthening of this variable.

The implementation of the escape room was carried out with university students from the health sector, which is important considering that some of these professionals will work directly with people affected by SMI, and their expectations and beliefs will affect the recovery of their patients [26]. Another aspect that should be highlighted is that professionals who work in the field of mental health continue to have negative beliefs, paternalistic attitudes, and even restrictive and directive attitudes on occasions [27]. These can have repercussions not only on recovery but also on the patients’ own perception of themselves, causing them to label themselves in a stigmatizing way, which is known as self-stigma [28].

In contrast, it has been proven how the narrative of the story and immersion in the activity encourage the re-elaboration of value judgments that alter the beliefs and attitudes of people with SMI. Escape rooms have already been tested as active methodological tools that promote motivation and student commitment toward learning [10,29], obtaining good results when they have been applied. Similar studies [30-32] have found that it is possible to reduce stigma through other methodologies, and the interesting aspect of this study is that it is able to raise awareness through a playful activity that is easy to implement and replicate.

As for other findings, a variable found to influence the results is whether the person has a family member with mental health problems. When a person has a family member with this type of difficulty, we can observe that the stigma is lower and possibly owes to the greater knowledge and direct contact that an individual has around these problems, which is fundamental in the reduction of stigma [33].

### Limitations

This study has several limitations. First, the sample is relatively small, it comes from 2 universities, and it is not representative of the general population. Furthermore, the sample of university students included only individuals from nursing schools. This may undermine the generalizability of the results and limit the interpretation of the effect size. Second, the specific characteristics of the participants and the possible confounding factors that may influence the results, such as their sociocultural origin, their level of knowledge, and their desire to work with patients with SMI, were not evaluated; the only exception was whether they had family members with SMI, in which case they were evaluated. Finally, another limitation was not conducting a reevaluation months after the intervention to determine whether the changes achieved in the experimental group could be perpetuated over time.

### Conclusions

Mental health awareness is a very important subject for the proper professional development of health care students. It is the responsibility of university teachers to prepare students to learn, understand, and know how to work with their patients. The lack of theoretical knowledge and stigma toward people with SMI can contribute to discrimination toward this group, which, in turn, can influence their self-esteem and quality of life. Therefore, it is necessary to implement new strategies in university education to improve the knowledge and awareness of mental disorders. The integration of playful strategies such as escape rooms can be of great interest because of their immersive and motivating capacity.

*Without Memories*, a web-based escape room where the narrative is about a character with SMI, is an effective tool to promote awareness around mental health because it favors learning by discovery and playful challenges through attractive dynamics and mechanics, which can encourage students to learn about SMI and be more empathetic toward these people.

Future studies could use this escape room not only in nursing degree studies but also in other degrees related to health and community education to promote awareness among other professionals who also have direct contact with people with SMI. Furthermore, future research could assess whether the results on the impact of playful strategies for mental health awareness vary because of other sociodemographic factors. Finally, the duration of the escape room *Without Memories* is very limited, and although this may have benefits for its replication, there is a need to plan future awareness programs through playful strategies of longer duration to evaluate whether they can achieve a greater impact on motivation, acquisition of knowledge, and awareness.

## Appendix

Multimedia Appendix 1 CONSORT-EHEALTH checklist (V 1.6.1).

## Abbreviations

AQ: Attributional Questionnaire
SMI: severe mental illness
